# Genetic diversity of *Plasmodium vivax *isolates from Azerbaijan

**DOI:** 10.1186/1475-2875-3-40

**Published:** 2004-11-09

**Authors:** Marie Claude Leclerc, Michela Menegon, Alexandra Cligny, Jean Louis Noyer, Suleyman Mammadov, Namig Aliyev, Elkhan Gasimov, Giancarlo Majori, Carlo Severini

**Affiliations:** 1 UR IRD 165, Génétique et Evolution des Maladies Infectieuses, UMR CNRS/IRD 2724, 911 Avenue Agropolis, BP 64501, 34394 Montpellier cedex 5, France; 2 Department of Infectious, Parasitic and Immunomediated Diseases, Instituto Superiore di Sanità, Viale Regina Elena 299, Rome, Italy; 3 CIRAD UMR 1096/PIA, TA40/03, Avenue Agropolis, F-34398 Montpellier, France; 4 Parasitology Department, Republican Center of Hygiene and Epidemiology, Baku, Azerbaijan; 5 National Research Institute of Medical Prevention, Baku; 6 WHO/AZE, Baku, Azerbaijan

## Abstract

**Background:**

*Plasmodium vivax*, although causing a less serious disease than *Plasmodium falciparum*, is the most widespread of the four human malarial species. Further to the recent recrudescence of *P. vivax *cases in the Newly Independent States (NIS) of central Asia, a survey on the genetic diversity and dissemination in Azerbaijan was undertaken. Azerbaijan is at the crossroads of Asia and, as such, could see a rise in the number of cases, although an effective malaria control programme has been established in the country.

**Methods:**

Thirty-six *P. vivax *isolates from Central Azerbaijan were characterized by analysing the genetic polymorphism of the circumsporozoite protein (CSP) and the merozoite surface protein 1 (MSP-1) genes, using PCR amplifications and amplicons sequencing.

**Results:**

Analysis of CSP sequences showed that all the processed isolates belong to the VK 210 type, with variations in the alternation of alanine residue (A) or aspartic acid residue (D) in the repeat motif GDRA(A/D)GQPA along the sequence. As far as MSP-1 genotyping is concerned, it was found that the majority of isolates analysed belong to Belem and Sal I types. Five recombinant isolates were also identified. Combined analysis with the two genetic markers allowed the identification of 19 plasmodial sub-types.

**Conclusion:**

The results obtained in the present study indicate that there are several *P. vivax *clones circulating in Azerbaijan and, consequently, a careful malaria surveillance could be of paramount importance to identify, at early stage, the occurrence of possible *P. vivax *malaria outbreaks.

## Introduction

*Plasmodium vivax *is the most widely distributed human parasite, with an estimated burden of 70–80 million cases annually [1]. In some parts of the world (Asia, South America), it is the most prevalent form of the four human malarial parasites. Although it causes a less severe disease than *Plasmodium falciparum*, being rarely lethal, *P. vivax *affects the working capacity of the population and the lack of efficient drug distribution favors the onset of drug resistant strains [2,3]. Imported malaria is an increasing health problem in Western Europe, where about 6,500 cases are reported annually in Germany, France, Italy and the United Kingdom [4]. Although *P. falciparum *infections account for the majority of cases (64%), *P. vivax *is responsible for an additional 23% [4]. Presence in this area of residual anopheline populations susceptible to *P. vivax *infection represents a permanent risk for the occurrence of *P. vivax *indigenous malaria cases, as recently occurred in central Italy [5,6]. Since 1970, malaria had been eradicated in central Asia, except for some residual foci in two countries belonging to the Newly Independent States (NIS), i. e. Azerbaijan and Tajikistan (WHO, Regional Office for Europe, unpublished document). At the beginning of the 1990s, the situation changed dramatically due to the re-emergence of malaria in the NIS area and especially in Tajikistan, where at present an epidemic is still in progress [7,8]. In these countries, the existing state of the primary health care system is extremely precarious, especially in rural areas and in small villages. Malaria is a common disease, which can easily re-establish itself when a lack of control occurs.

In comparison with *P. falciparum*, molecular studies of the genetic diversity and dissemination of *P. vivax *are scanty. Recently, 33 polymorphic tandem repeats (TRs) of *P. vivax *and a *P. vivax *polymorphic microsatellite have been identified and shown to be useful in population studies [9,10]. The merozoite surface protein 3α (MSP3-α) gene also seems to be a good candidate for studying the genetic diversity of *P. vivax *populations, since PCR-RFLP products indicate the presence of up to 13 alleles [11,12]. However, the circumsporozoite protein (CSP) and merozoite surface protein 1 (MSP-1) genes still remain the most studied molecular markers in genetic epidemiological surveys carried out in *P. vivax *endemic areas.

In the frame of a malaria research project funded by the European Commission, a molecular study was undertaken in Azerbaijan, aimed at collecting information on the genetic make-up of *P. vivax *natural populations present in this endemic country. For this purpose the extent of polymorphism of CSP and MSP-1 genes were analysed in parasite isolates from five localities of central Azerbaijan by using PCR amplification and sequencing.

## Materials and Methods

### Study area and samples collection

Azerbaijan covers an area of 29.540 Km^2^, with a populations of approximatively 2,5 millions. The climate is typically continental with an average temperature between 12 and 15°C and a rainfall between 200 and 600 mm per year. Climatic and agro-ecological conditions of this area make the environment favourable to mosquito vectors breeding. The major malaria vector is *Anopheles sacharovi *that breeds preferably in lakes, swamps, irrigation canals and pools. Although it prefers well-oxygenated water, it is known to tolerate moderate salty water. Other anopheline species found in this area are *A. maculipennis*, *A. subalpinus*, *A. superpictus *and *A. hyrcanus*, all of which are considered secondary vectors of malaria transmission [13]. Malaria transmission occurs in Azerbaijan mainly from June to October. In the last years, number of malaria cases showed a negative trend, accounting for 610 cases in 2000, 418 cases in 2001, 203 cases in 2002.

**Figure 1:**
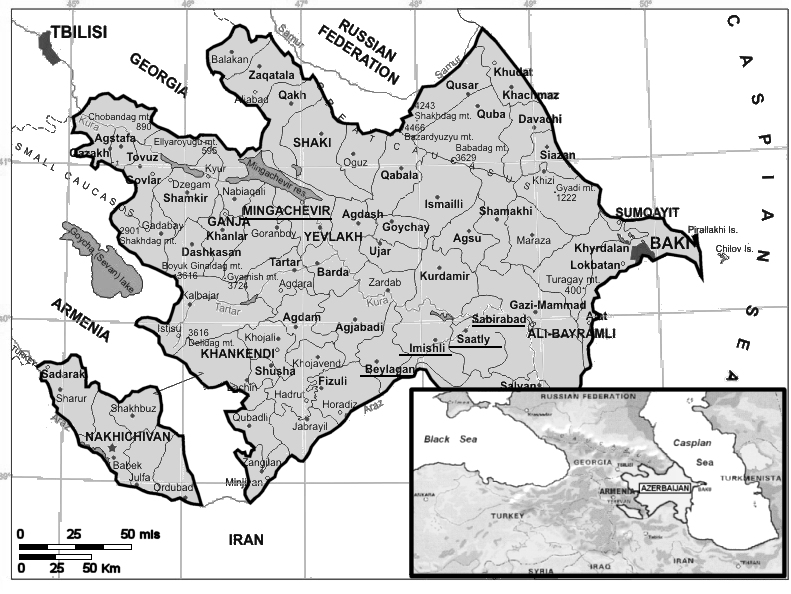
Map of central Azerbaijan showing localities (underlined names on the map) included in the present study.

During summer 2002, a malaria epidemiological survey was performed in central Azerbaijan, in the frame of a Malaria Surveillance Programme launched in year 2001 by the Ministry of Health in collaboration with WHO. Active case detection was carried out in five districts included in previously identified sentinel sites, namely Mingaçevir, Beylagan, Imisli, Saatli and Sabirabad. A map of Azerbaijan with the study area is shown in Figure 1.

All individuals who visited the district health centres or were found in villages with a history of recent fever and no history of travel in the past few months were considered. In this context, a total of 36 infected individuals with positives blood smears at the microscopic examination, collected between August and September 2002, were selected for the genetic study. The age of patients ranged from 6 to 78 years and parasitaemia varied from 288 to 12,800 parasites/μl. For the molecular analysis, a blood sample of about 1 ml was taken from by venipuncture before drug treatment was given. Patients or the guardians of children were informed about the study. According to the international rules for research involving human subjects, any information which would identify a participant was removed in order to keep each sample processed anonymous. Number of samples for each district is shown in Table 1.

**Table 1:** Geographic origin of Azerbaijan isolates with the corresponding MSP-1 and CSP characteristics identified in the present study.

**ISOLATE NAMES**	**MSP-1**			**CSP**	
	
	Genotype	No. polyQ	Sub-type	Genotype	Sub-type
Bey1	Belem	21	G	VK210	4
Bey2	Belem	21	G	"	4
Bey4	Sal1	-	O	"	5
Bey7	Belem	21	G	"	8
Bey14	recombinant	19	S	"	2
Im3	Belem	21	G	"	4
Im5	Belem	21	G	"	4
Im8	Sal1	-	H	"	1
Im9	Belem	21	G	"	4
Im10	Belem	21	F	"	4
Im11	Belem	21	G	"	4
Im12	Belem	21	C	"	4
Im14	Belem	21	D	"	4
Im15	Belem	21	G	"	4
Min1	Belem	21	G	"	4
Min3	Belem	21	G	"	4
Min6	Sal1	-	L	"	3
Min7	recombinant	19	S	"	2
Min8	Sal1	-	I	"	2
Min9	Belem	21	G	"	4
Min10	Sal1	-	P	"	6
Sat2	Belem	21	G	"	4
Sat3	Belem	21	F	"	4
Sat5	recombinant	18	U	"	5
Sat7	recombinant	18	U	"	5
Sat11	recombinant	12	T	"	5
Sab1	Belem	21	G	"	7
Sab2	Belem	21	A	"	4
Sab4	Belem	21	B	"	4
Sab6	Sal1	-	M	"	4
Sab7	Belem	21	G	"	4
Sab8	Belem	21	G	"	4
Sab10	Sal1	-	M	"	4
Sab12	Belem	21	G	"	4
Sab13	Sal1	-	R	"	4
Sab15	Sal1	-	Q	"	4

### DNA preparation

Plasmodial DNA was extracted from 200 μl of each infected blood sample using QIAamp DNA blood kit following the manufacturer's instructions (Qiagen, CA).

### Circumsporozoite (CSP) marker analysis

The CSP gene was amplified for the most part of samples using PV5 and PV6 primers [14]. Samples that did not provide good PCR products with this set of primers were processed a second time by using CSP-A2 [15] as forward primer and PV6bis (5'-CACAGGTTACACTGCATGGAGT-3') as original reverse primer. PCR amplification was performed in a reaction mixture of 50 μl containing the parasite DNA, 1x reaction buffer, 2.5 mM MgC1_2_, 80 μM of each deoxynucleotide triphosphate, 6 pmol of each primer and 1.3 U of *Taq *polymerase (Promega, Madison, USA). The PCR programme was: denaturation at 94°C for five minutes; 34 cycles of one minute at 94°C, one minute at 54°C and two minutes at 72°C. The PCR products were separated using electrophoresis on a 1.5 % NuSieve gel and the band of interest was cut out and purified using the QIAquick PCR purification Kit (Qiagen). The purified product was sequenced in both directions using an ABI-PRISM 373 sequencer. Nucleotide or amino acid sequences were aligned first using the CLUSTAL X programme [16] with manual editing and adjustments made using the MUST package [17]. The ExPASy Molecular Biology Server  was used to convert nucleotide sequences into amino acid sequences. The GenBank accession numbers of the eight sub-types of VK210 type are from AY792359 to AY 792366.

### Merozoite surface protein 1 (MSP-1) marker analyses

A portion of the MSP-1 gene (the region encompassing the interspecies conserved blocks ICB5 and ICB6) was amplified using a nested PCR with, respectively, the two outer primers A5 and A6 [18] and the two inner primers MSP1N1 forward and MSP1N2 reverse [6]. The first round of amplification was performed in a reaction mixture of 50 μl containing parasite DNA, 1x reaction buffer, 2.5 mM MgC1_2_, 200 μM of each deoxynucleotide triphosphate, 30 pmol of each primer and 2.5 U of *Taq *polymerase (Promega, Madison, USA). For the second round, 10 μl of the first amplification product was added to a fresh PCR mixture with 30 pmoles of each inner primer. The thermal profile was: denaturation at 94°C for five minutes; 35 cycles of 94°C for one minute, 60°C for one minute and 75°C for three minutes. All nested-PCR products were purified by Microcon-PCR (Millipore), following the manufacturer's instructions and sequenced in both directions at the MWG Biotech. The results were analysed by means of Omiga 2.0 (ACCELRYS, Cambrige) and Mega 2 (S. Kumar, K. Tamura, M. Nei and Pennsylvania State University) computer programmes. The GenBank accession numbers of the 36 nucleotide sequences from *P. vivax *isolates are from AY789657 to AY789692.

### Distance analyses

The aligned nucleotide sequences of CSP were converted to a distance matrix (% of differences) using the Net algorithm of the MUST package [17]. The dendrogram was generated using the neighbour-joining method [19]. Bootstrap proportions were used to assess the robustness of the tree with 1,000 bootstrap replications [20].

MSP-1 and CSP data were analysed using the Cavalli-Sforza distance [21] from Genetics v.4.01 package. The dendrogramme was generated using the neighbour-joining method [19]

## Results

### CSP marker

**Figure 2:**
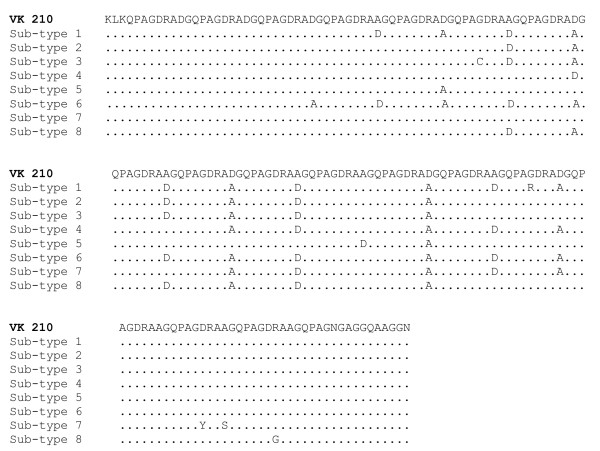
Amino acid sequence alignment of eight CSP sub-types found from 36 Azerbaijan *P. vivax *isolates with that of VK210 type (Accession No. M28745). ^a ^Imi 8; ^b ^Bey 14, Min 7, 8; ^c ^Min 6; ^d ^Bey 1, 2, Imi 3, 5, 9, 10, 11, 12, 14, 15, Min 1, 3, 9, Sat 2, 3 Sab 2, 4, 6, 7, 8, 10, 12, 13, 15; ^e ^Bey 4, Sat 5, 7, 11; ^f ^Min 10; ^g ^Sab 1; ^h ^Bey 7.

**Figure 4:**
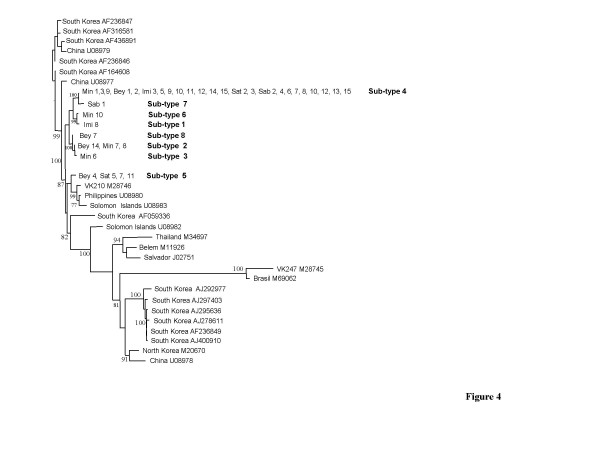
Distance tree (built with the neighbor-joining method) inferred from 443 nucleotide positions and 264 variable sites of CSP gene. Numbers on the branches indicate bootstrap proportions (1000 replicates); only bootstrap values above 70 % are displayed on the tree.

CSP sequences obtained from 36 Azerbaijan *P. vivax *isolates were found to belong to the VK210 type [22]. The isolates tested displayed variations in the peptide repeat motifs GDRA(A/D)GQPA with different alternations of non-synonymous codons GCT or GAT, respectively, coding for alanine (A) and aspartic acid (D) (Figure 2). All our sequence types had the same three repeat units (GDRAAGQPA) at the 3' end, identical to that of the VK210 type. Furthermore four non-synonymous mutations were found, one being the RDRADGQPA variant (sequence named in the present study as sub-type 1), already described in North Korean and Chinese isolates [23]. In summary, eight different sub-types of VK 210 were observed (Figure 2 and Figure 4). Among all 36 azeri isolates analysed, 24 isolates were found to have identical sequence (sub-type 4, Table 1 and Figure 4). In particular, the Beylagan (n = 5) and Mingaçevir (n = 7) isolates appeared the most diversified since they displayed four and five different sub-types respectively. The Imishli (n = 9), Saatli (n = 5) and Sabirabad (n = 10) isolates only showed two different sub-types each-one. Figure 4 clearly shows that the genetic diversity of CSP is relatively small inside the Azerbaijan isolates when compared to the South Korean and Chinese isolates.

### MSP-1 marker

**Figure 3:**
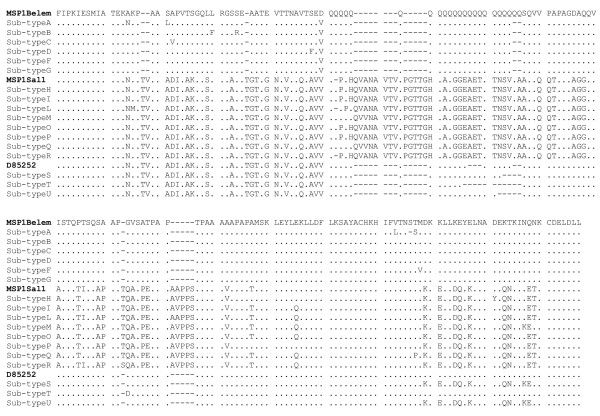
Amino acid sequence alignment of seventeen MSP-1 sub-types found from 36 Azerbaijan *P. vivax *isolates compared with that of MSPlBelem (Accession No. M60807), MSPlSal1 (Accession No. M75674) and recombinant type 3a (D85252). Classification of Azeri isolates according to the different types is shown in Table 1.

The majority of Azerbaijan isolates (Table 1) belong to either the Belem (22 isolates, all with the same poly-Q region of 21 repeats) or the Sal I (9 isolates) types already described [24]. Only 5 *P. vivax *isolates were identified as recombinant types. Isolate Satl1 (sequence named in the present study as sub-type T) could be ascribed to the type 3a (accession no. D85252, [25]), while isolates Bey 14 and Min7 (sub-type S) and isolates Sat5 and Sat7 (sub-type U) seem to be the result of recombinant events between the recombinant type 3a and Sal I. All the three recombinant types showed a different number of poly-Q repeats (Table 1). In addition to these sources of diversity, nucleotide substitutions could be observed, allowing the identification of 17 sub-types (Table 1 and Figure 3). The Imisli and Sabirabad districts appeared to be less diversified, accounting for five different genotypes for 9 isolates and six genotypes for 10 isolates, respectively. Finally, Saatli district was found to have the greatest variability, with four different genotypes for 5 isolates.

### Combined analysis between the two markers

**Figure 5:**
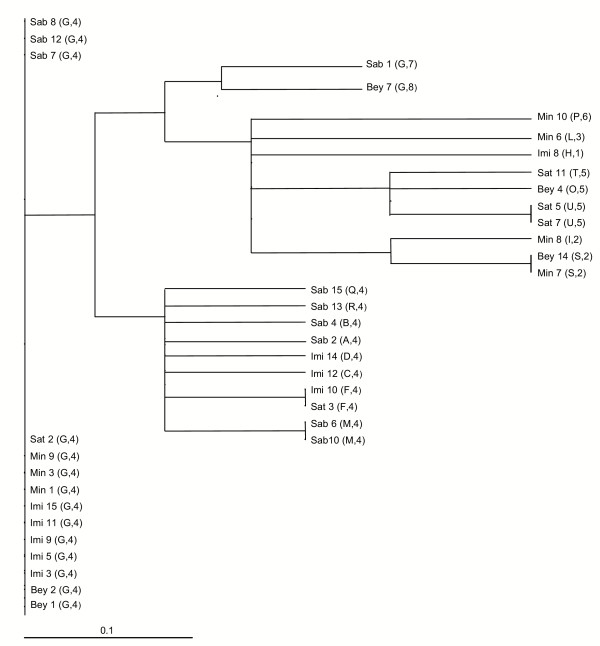
Neighbour-joining tree from the MSP-1 and CSP data (results in parenthesis) reflecting the relationships between the Azerbaijan *P. vivax *isolates.

By combining the results of genotyping obtained by CSP and MSP-1, 19 *P. vivax *sub-types (Figure 5) were identified as circulating in the central region of Azerbaijan. The sub-type named G/4 with the greatest representation (n = 14 isolates), was detected in all districts investigated. Genotypes identified as M/4 and U/5 were observed twice in the districts of Sabirabad and Saatli, respectively. Genotype F/4 was detected once in Imisli and Saatli districts, as was for genotype S/2, detected once in Beylagan and Mingacevir districts.

## Discussion and conclusions

For CSP, the main variations already reported in the literature consist of two variant sequences, VK210 and VK247 that show a variable number of repeat units, GDRA(A/D)GQPAA and ANGAGNQPG, respectively, with some variant positions within the sequence [22,23]. These two variants have a worldwide distribution, and locally their distribution have been also correlated with climatic gradients or with the Anophelines vector specificity [26]. Studies carried out in some Asian endemic countries, i.e. South and North Korea, China, the Philippines, the Solomon Islands and Thailand [27,15,28,12] suggest that CSP has a limited value as a molecular marker for genetic variability when used alone. In the present study, all the analysed *P. vivax *isolates from Azerbaijan were found to belong to the type VK210 and they constitute a group of eight CSP sub-types that closely linked in the dendrogramme shown in Figure 4. Differently from what reported by other Authors [28] who showed that the CSP sequence analysis allows detecting the geographic origin of plasmodial isolates, our results did not support its use for tracking the geographic origin of Azeri isolates since we dealt with a limited number of samples studied. No genotype association with particular sampling districts was observed since, for example, the most common sub-type identified (G/4) was present in all five districts.

Our results confirmed that MSP-1 is a good polymorphic marker. In particular, the region of the gene known to be highly polymorphic and discriminative between the Belem and Sal I types was analysed [23]. A variable poly-glutamine (poly-Q) region is characteristic of the Belem type and represents the principal source of genetic diversity of this marker. Moreover, a poli-Q region is the recombination site between the two types Belem and Sal I and, as shown in the literature, interallelic recombinations between the two types are frequent [25]. The total genetic diversity observed when including the nucleotide substitutions is relatively important taking into account the low endemicity of studied area. Similar results were observed in Southeastern Iran and in Thailand [29,12], low endemic countries for *P. vivax *malaria as well, where the authors detected the two types Belem and Sal I, together with several recombinant types. In particular, in the study carried out in Iran by Zakeri *et al.*, the analysis of MSP1 genetic diversity on a total of 16 plasmodial isolates leaded to the identification of 14 genetic sub-types. It is worth noting that such a high level of diversity is probably due to the small sample size.

Our results show 17 genetic sub-types on a total of 36 isolates analysed and a quite high MSP-1 polymorphism also in Azerbaijan. As suggested in other studies [12] and also reported by Zakeri *et al.*, it is possible to speculate that the observed genetic diversity could be also explained considering the studied area, i.e. central Azerbaijan, as transit road of the country and also of neighboring Asian country, where the circulating *P. vivax *populations show considerable MSP-1 genetic diversity. However, further studies aimed at collecting more information about people moving within the whole country and to closer countries are needed to verify the above hypothesis. The combined analysis of CSP and MSP1 sequence polymorphism has led to the identification of a total of 19 *P. vivax *sub-types, confirming that the simultaneous use of more than one genetic marker in this kind of study enhances the knowledge of genetic diversity existing in the parasite populations. The results of the current study show the circulation of multiple plasmodial clones in the studied area thus leading to the conclusion that malaria surveillance activities must be maintained in Azerbaijan in order to avoid serious disease outbreaks in the future.

The understanding of the polymorphism extent in surface antigens as CSP and MSP-1 and the resulting genetic diversity in *P. vivax *field populations could help in implementing malaria control activities being a crucial step for the development of a malaria vaccine.

## Authors' contributions

S. Mammadov, N. Aliyev, E. Gasimov were involved in field collection of blood samples and microscopy examinations. M.C. Leclerc and A. Cligny did the CSP sequence analysis and M. Menegon did the MSP-1 sequence analysis. M.C. Leclerc and J.L. Noyer did the distance analyses. M.C. Leclerc wrote the report with major contributions of C Severini, M Menegon and G. Majori. G. Majori coordinated the field activities carried out in Azerbaijan. C. Severini, as scientific coordinator of the VIVAXNIS project mentioned below, got the financial support.
